# Evaluation of methods to purify virus-like particles for metagenomic sequencing of intestinal viromes

**DOI:** 10.1186/s12864-014-1207-4

**Published:** 2015-01-22

**Authors:** Manuel Kleiner, Lora V Hooper, Breck A Duerkop

**Affiliations:** Department of Immunology, University of Texas Southwestern Medical Center, Dallas, TX 75390 USA; The Howard Hughes Medical Institute, University of Texas Southwestern Medical Center, Dallas, TX 75390 USA; Current address: Department of Geoscience, University of Calgary, Calgary, AB T2N 1 N4 Canada

**Keywords:** Virus metagenomics, Viral metagenomes, Virus-like particles, Microbiome, Bacteriophage, CsCl density gradient

## Abstract

**Background:**

Viruses are a significant component of the intestinal microbiota in mammals. In recent years, advances in sequencing technologies and data analysis techniques have enabled detailed metagenomic studies investigating intestinal viromes (collections of bacteriophage and eukaryotic viral nucleic acids) and their potential contributions to the ecology of the microbiota. An important component of virome studies is the isolation and purification of virus-like particles (VLPs) from intestinal contents or feces. Several methods have been applied to isolate VLPs from intestinal samples, yet to our knowledge, the efficiency and reproducibility between methods have not been explored. A rigorous evaluation of methods for VLP purification is critical as many studies begin to move from descriptive analyses of virus diversity to studies striving to quantitatively compare viral abundances across many samples. Therefore, reproducible VLP purification methods which allow for high sample throughput are needed. Here we compared and evaluated four methods for VLP purification using artificial intestinal microbiota samples of known bacterial and viral composition.

**Results:**

We compared the following four methods of VLP purification from fecal samples: (i) filtration + DNase, (ii) dithiothreitol treatment + filtration + DNase, (iii) filtration + DNase + PEG precipitation and (iv) filtration + DNase + CsCl density gradient centrifugation. Three of the four tested methods worked well for VLP purification. We observed several differences between methods related to the removal efficiency of bacterial and host DNAs and biases against specific phages. In particular the CsCl density gradient centrifugation method, which is frequently used for VLP purification, was most efficient in removing host derived DNA, but also showed strong discrimination against specific phages and showed a lower reproducibility of quantitative results.

**Conclusions:**

Based on our data we recommend the use of methods (i) or (ii) for large scale studies when quantitative comparison of viral abundances across samples is required. The CsCl density gradient centrifugation method, while being excellently suited to achieve highly purified samples, in our opinion, should be used with caution when performing quantitative studies.

**Electronic supplementary material:**

The online version of this article (doi:10.1186/s12864-014-1207-4) contains supplementary material, which is available to authorized users.

## Background

In the last decade there has been an increasing appreciation that the intestinal microbiota of mammals has a strong influence on host metabolism, physiology and health [1-3]. Metagenomics has enabled large scale studies of these complex microbial communities in the intestine revealing both a qualitative and quantitative picture of the phylogenetic and functional diversity of intestinal microbes [4-6]. The majority of intestinal metagenomic studies have focused on the bacterial component of the microbiota during states of health and disease. In recent years viruses, including bacteriophages (phages), from the mammalian intestine have started to receive much attention [7-9]. The contribution of phages to intestinal microbiota ecology and their potential effects on the mammalian host are just beginning to be elucidated [10-14].

To unravel the influence of viruses, and particularly bacteriophages, on microbiota ecology and animal host physiology and health, methods allowing quantitative comparison of virus diversity, abundance and function across samples are needed. Sequencing of viromes (metagenomes of virus-like particles, VLPs) is one method that enables such quantitative comparisons [8,14,15]. A crucial step for virome sequencing is the purification of VLPs from fecal samples. VLP purification is necessary for the following two reasons. First, viruses often have very small genomes compared to bacteria and host derived DNAs. Therefore, viral DNA represents a small percentage of the total DNA in a metagenomic sample [15]. This leads to a proportionally low representation of viruses in the obtained sequencing information if complete microbiomes are sequenced. Second, many phages in the intestinal microbiota are integrated into the genomes of their bacterial hosts as dormant lysogenic prophages [15]. By isolating VLPs it is possible to distinguish integrated prophage genomes from phage genomes that are associated with viral particles. While methods for VLP purification from environments such as seawater have been well-analyzed [16], methods for isolating and purifying intestinal viromes are understudied. In some environments such as the open ocean viral density is low and VLP concentrating methods such as tangential flow filtration or FeCl_3_ precipitation have to be used in addition to purification methods to obtain a sufficient density of VLPs for sequencing [16]. In intestinal samples, however, viruses are already highly concentrated and thus additional VLP concentration is not necessary [15].

Recent studies of intestinal viromes have used several different methods to purify VLPs from fecal samples and to prepare the DNA for sequencing [14,15]. While the effects of DNA amplification, library preparation and sequencing method on virus metagenomes have been investigated in great detail [17-21], a critical evaluation of methods for the purification of VLPs from intestinal content or feces has, to our knowledge, not yet been conducted.

Since phages show great variability in terms of shape, size, buoyant density, resistance to chemical and mechanical stressors, and nucleic acid content [22,23], it can be expected that the method of purification will strongly influence the degree to which specific phages and other viruses are retained in purified samples. For example, one method that has been widely used for VLP purification is cesium chloride (CsCl) density gradient centrifugation, which purifies phages within specific density ranges and discriminates against phages that fall outside of a specified density.

The aim of our study was to evaluate methods for VLP purification from fecal samples which can be applied to samples in a reproducible and quantitative manner. These methods should be amenable to large sample numbers in parallel to enable the use of replicates. Furthermore, the methods should permit quantitative comparisons of intestinal viromes across multiple individuals and varying treatment groups. To assess the effects of purification methods on VLP recovery we used an artificial microbiota of known composition. This artificial microbiota contained six phages and two bacterial species for which complete genome sequences were available.

## Results and discussion

We used an artificial intestinal microbiota sample consisting of germ-free mouse feces containing six phages (P22, T3, T7, ɸ6, M13 and ɸVPE25) and two bacterial strains (gram-positive: *Listeria monocytogenes* EGD-e and gram-negative: *Bacteroides thetaiotaomicron* VPI5482). Phages P22, T3, T7, and ɸVPE25 represent double-stranded DNA (dsDNA) genomes, M13 has a linear single-stranded DNA (ssDNA) genome, and ɸ6 has a segmented double-stranded RNA (dsRNA) genome. The phages were added in equal numbers and the total number of phage particles (plaque forming units, PFU) equaled the total number of bacteria (colony forming units, CFU) added to the sample (see Methods section for details). The two bacterial strains were added to the sample at a 1:1 ratio relative to each other.

**Figure 1:**
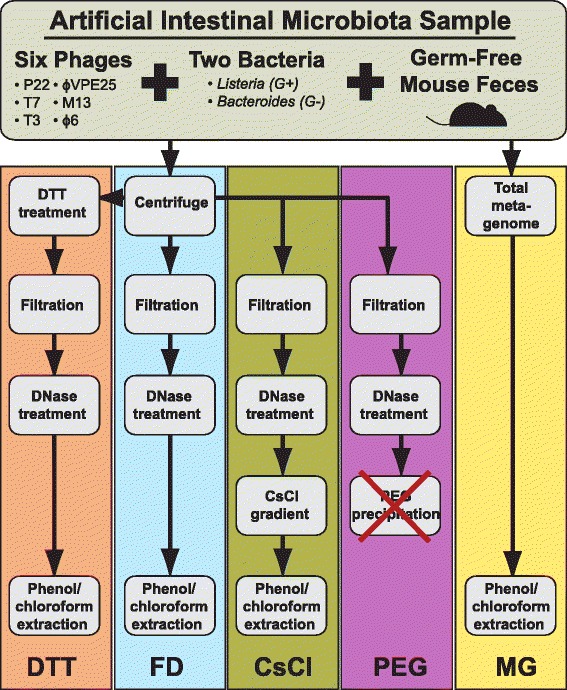
**Schematic diagram of VLP purification methods.** An artificial intestinal microbiota sample was generated by the addition of six phages and two bacterial strains to germ-free mouse feces. Upon homogenization of the mixture, the sample was split into 10 equal subsamples. Two subsamples were immediately set aside for total DNA isolation (metagenome). The remaining 8 samples were centrifuged. Upon completion of centrifugation, two samples were treated with 6.5 mM dithiothreitol (DTT). All eight samples were filtered to remove bacteria and particulates and treated with DNase. Two samples were loaded onto a CsCl gradient and phages were banded as described in Methods. The PEG precipitation failed due to the formation of a viscous mass that impaired PEG removal by buffer exchange. After collection of the phages from the CsCl gradients, all 8 samples were extracted for total nucleic acids using phenol/chloroform extraction. G+, Gram-positive; G-, Gram-negative.

We tested and evaluated four different methods to purify phages from mouse feces for quantitative metagenomic studies (i.e. allowing for cross-comparison of relative abundances between samples). The four methods, which we designed based on standard protocols used for virus purification [15,16,23-25], included: (i) removal of microbial cells by filtration + removal of free DNA by DNase digestion (FD), (ii) dithiothreitol treatment to degrade fecal mucus + filtration + DNase (DTT), (iii) filtration + DNase + condensation mediated phage particle precipitation with polyethylene glycol (PEG) and (iv) filtration + DNase + CsCl density gradient centrifugation to purify phages based on density (CsCl) (Figure 1, more details in methods section). A fifth treatment group consisted of the total metagenome (MG) of the original, unpurified sample. Since our study focused on the effects of phage purification methods, we used identical DNA extraction and library preparation steps for all samples and processed them in parallel.

To test the purification methods we divided the artificial microbiome sample into ten subsamples of equal mass (0.27 g each). Eight subsamples were used to carry out the purification methods (FD, DTT, PEG and CsCl) in duplicate. The remaining two subsamples were used for extraction of the total metagenome (MG). We will use the following abbreviations for the replicate metagenomes throughout the article: FD1 and FD2 (filtration + DNase), DTT1 and DTT2 (DTT + filtration + DNase), CsCl1 and CsCl2 (filtration + DNase + CsCl), MG1 and MG2 (complete metagenome). During purification, the PEG method failed due to the formation of a viscous high molecular weight compound upon addition of the PEG to the sample filtrate. This precipitate prevented the subsequent removal of PEG by buffer exchange and these samples could no longer be processed as desired (Figure 1). In the future, the PEG method could likely be improved by removing PEG by chloroform extraction instead of buffer exchange, however, several virus groups are sensitive to chloroform and would thus be lost during PEG extraction (see e.g. [23] for a list of virus sensitivities). All eight remaining samples were subjected to paired-end sequencing on an Illumina HiSeq 2500 sequencer generating ~14 million paired-end reads per sample.

### DNA recovery

All three working purification methods (FD, DTT and CsCl) yielded <10% of the DNA amount extracted from the MG samples (Table 1). MG1 yielded 636 ng and MG2 yielded 459 ng of DNA. The CsCl samples had the lowest yield, approximately 20 ng. Yields in the FD samples were around 40 ng, while the DTT samples were intermediate (DTT1: 29 ng, DTT2: 34 ng). Most of the reduction in DNA between the MG samples and the purified samples is likely due to the removal of bacterial and mouse DNA during sample purification.

**Table 1:** **Method overview/summary**

	**FD**	**DTT**	**CsCl**	**MG**
**Purification steps**	**Filtration + DNase**	**DTT + Filtration + DNase**	**Filtration + DNase + CsCl centrifugation**	**Total metagenome of unpurified sample**
DNA recovered in (ng in sample 1, ng in sample 2)	42, 44	29, 34	21, 19	636, 459
Sample throughput^a^	15-20	15-20	6-8^d^	N/A
Total duration of protocol (days)^b^	1	1	2	N/A
Hands on time (hours)^c^	6	6	10	N/A
Special equipment needed	No	No	Ultracentrifuge	N/A
Intra-method reproducibility	High	High	Medium	N/A
Biases against specific phage	Weak	Weak	Strong	N/A
Removal efficiency of mouse DNA	High	High	Very high	N/A
Removal efficiency of bacterial DNA	Very high	Very high	Very high	N/A

^a^:Number of samples that can be processed by one person in parallel; ^b^:Duration of the respective purification protocol from fecal sample to purified VLPs, DNA extraction time not included; ^c^:Hands on time needed for the number of samples that can be processed by one person in parallel (above); ^d^:Sample number limited by rotor size of ultracentrifuge and number of density gradients that can be set up in parallel in a reasonable amount of time.

### Purification efficiency, reproducibility and biases of the purification methods based on read coverage

**Figure 2:**
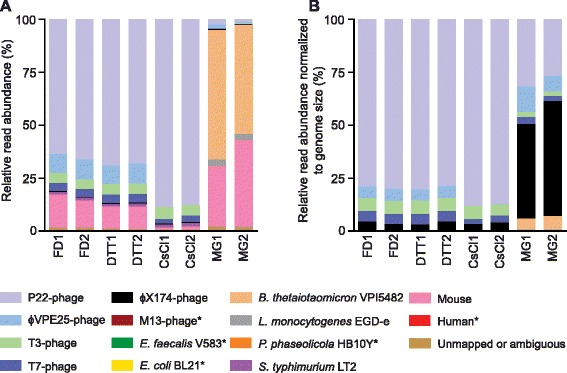
**Read abundances from artificial intestinal microbiota samples following VLP purification or whole metagenome processing. (A)** Relative read abundances (detailed data in Additional file 1: Tables S1 and S3-S10) **(B)** Relative read abundances after normalization to genome sizes (Detailed data in Additional file 1: Tables S2-S10). FD, Filtration + DNase – replicates 1&2; DTT, DTT + Filtration + DNAse – replicates 1&2; CsCl, Filtration + DNase + CsCl gradient – replicates 1&2; MG; Total metagenome (no purification procedure) – replicates 1&2. *Denotes organism read abundances with bars too small to be seen in the figure.

To evaluate the purification methods we mapped the reads from the eight metagenomes against a set of reference sequences consisting of the genomes of the input phages and bacteria plus the genomes of expected contaminants such as mouse, human and the ɸX174 phage that is used as an internal control during Illumina sequencing (Figure 2, Additional file 1: Tables S1-S10). More than 97% of all reads mapped unambiguously to one of these reference genomes. The remaining reads either mapped ambiguously to several of the reference genomes or did not map to any of the reference genomes. The small number of reads that did not map to any of the reference genomes indicates that the content of unknown DNA in the samples was small.

In the following we will use differences in relative read abundances between metagenomes as an estimate of differences in relative DNA amounts in the sample. Also, for simplicity, we will refer to sequencing reads mapping to the genome of a specific organism or organism group, as < organism > reads e.g. mouse reads for reads mapping to the mouse genome and phage reads for the reads mapping to the genomes of all added phages (excluding the ɸX174 internal Illumina control).

All three purification methods (FD, DTT and CsCl) led to an average increase of phage reads of more than 20-fold. While phage reads account for <5% of all reads in the MG samples, they account for >80% of all reads in the purified samples (Figure 2, Additional file 1: Table S1). The highest percentage of phage reads was achieved in the CsCl samples, followed by the DTT samples and then the FD samples. In terms of purification efficiency the CsCl method is the most efficient at removing mouse DNA contamination, however, it has other drawbacks that need to be considered (see below and Table 1).

Intra-method reproducibility was observed for the FD and DTT methods, as judged by the relative read abundances of the four phages for which good read coverage was achieved (P22, ɸVPE25, T3 and T7). The intra-method ratios of relative read abundances for specific phages were between 0.92 and 1.03, which is close to the theoretical optimum of 1 (Table 2). In contrast, much higher variability was observed between the two CsCl replicates, where ratios between 0.71 and 1.14 were observed (Table 2). This higher intra-method variability for the CsCl method may have been caused by the fraction collection, which was based on the protocol published by Thurber et al. [23]. The reproducibility might be improved by using specialized gradient-harvesting devices for gradient fractionation followed by careful evaluation of fraction densities [26,27].

**Table 2:** Intra- and between method variation based on read abundance ratios*

	**Intra-method variation**				**Between method variation** ^**a**^		
	**FD1/FD2**	**DTT1/DTT2**	**CsCl1/CsCl2**	**MG1/MG2**	**FD/DTT**	**DTT/CsCl**	**CsCl/FD**
Calculated with % read abundance							
M13	0.74	2.22	1.67	1.08	1.52	10.06	0.07
P22	0.96	1.01	1.01	1.73	0.95	0.78	1.36
ɸVPE25	0.94	0.92	0.75	2.33	0.99	125.23	0.01
T3	1.01	1.01	1.14	1.69	0.93	0.94	1.15
T7	1.03	1.00	0.71	1.88	0.94	1.57	0.68
Calculated with % read abundance normalized to genome size							
M13	0.75	2.24	1.63	0.73	1.60	11.20	0.06
P22	0.98	1.02	1.01	1.19	1.00	0.91	1.10
ɸVPE25	0.96	0.93	0.75	1.60	1.05	146.16	0.01
T3	1.03	1.01	1.14	1.16	0.98	1.09	0.93
T7	1.05	1.00	0.71	1.29	0.99	1.83	0.55

*Perfect reproducibility would result in ratios equal to one.

^a^:Read abundance ratios between methods were calculated using the average read abundance in each method.

As expected, variability between methods was higher than intra-method variability. The ratios of relative read abundances for specific phages between the FD and DTT methods were close to 1 indicating reproducible quantitative results between these methods. In contrast, some of the ratios between the CsCl method and the FD and the DTT methods deviated significantly from 1 indicating that different purification biases exist between these methods (Table 2). In particular, the read abundances of the ɸVPE25 and T7 phages were greatly reduced in the CsCl purified samples (Figure 2, Additional file 1: Table S1). We also observed that when read abundances were normalized to genome sizes, the ratios between the FD and DTT methods were even closer to 1 (Table 2, Additional file 1: Table S2). This can be explained by the fact that normalization of read abundance to genome size yields an estimate of relative genome copy number, which is less influenced by small changes in copy number of contaminating DNA originating from large genomes (e.g. mouse). Small changes in copy number of large genomes can influence read abundances significantly, because these genomes yield a read quantity proportional to their size during sequencing. Our results suggest that normalization of read abundance to genome size (if known) can improve between- sample and -method comparability.

Although all phages were mixed into the artificial microbiota sample in equal numbers (based on PFU count) their representation in the metagenomes differed greatly from an equal distribution. Since input number of phages should roughly correspond to the input genome copy number for each phage, the read abundances normalized to genome size should in theory be equal for all phages. Consequently, the ratios of these read abundances should be close to 1 within any given sample. However, even in the unaltered MG samples, this is not the case (Figure 2B, Table 3, and Additional file 1: Table S2). Both the P22 and the ɸVPE25 phages have much higher read abundances in the MG samples as compared to the T3, T7 and M13 phages. For the M13 phage this distortion in read abundance can be explained by its ssDNA genome (see below). For the other phages, there are three potential explanations for why the read abundances differ from expected read abundance in the MG samples. First, phage genome copy number could be misestimated by the PFU counting method. This method only counts viable phages that are able to infect and lyse their host. Non-viable VLPs or free phage DNA would not be considered in these measurements. It is common for phage lysates to contain a large number of non-viable VLPs, which contain nucleic acid, but are not able to produce plaques [28]. To check whether the unequal read abundances between the four dsDNA phages are caused by a misrepresentation of total VLPs by PFU counting we counted total VLPs by epifluorescence microscopy and compared them to the PFU counts (Additional file 1: Table S11). As expected the total VLP count was higher than the PFU count and the VLP/PFU ratios ranged from 4.8 to 8.2 for specific phages (Additional file 1: Table S11). However, based on this data the phage input numbers into the artificial microbiome sample are still close to the 1:1 ratio (less than two-fold difference between all phage input numbers), which we initially determined by PFU counting and thus the large observed differences in read abundances cannot be explained by differences between PFU and VLP counts. Interestingly, the P22 phage, which has the highest read abundance in all treatments, was put into the artificial microbiome sample in lowest number based on VLP counting. The unequal read abundances for phages in the MG samples could, however, be explained by free phage DNA, which would not be detected by either VLP or PFU counting. The fact that the read abundance ratios for the T7, T3 and ɸVPE25 are close to 1 in the FD and DTT samples suggests that the unequal read abundances for these phages in the MG samples were caused by free phage DNA that was removed during the purification procedure. Second, the DNA extraction method may extract phages with different efficiencies. However, since the T3, T7 and P22 phages are structurally similar (all three are members of the *Podoviridae*), this seems an unlikely explanation. Third, phage genomes can carry a variety of DNA modifications [29] that could lead to biases during sequencing library preparation. It was recently shown that some DNA modifications can lead to strong exclusion biases during Illumina library preparation [30].

**Table 3:** Purification method biases for specific phages*

	**FD**	**DTT**	**CsCl**	**MG**
Average % read abundance normalized to genome size				
M13	0.010	0.007	0.001	0.027
P22	79.605	79.695	87.830	29.338
ɸVPE25	5.564	5.310	0.036	9.749
T3	6.164	6.286	5.751	2.101
T7	4.879	4.923	2.691	2.974
Ratios of genome size normalized % read abundances				
P22/ɸVPE25	14.3	15.0	2417.5	3.0
P22/T3	12.9	12.7	15.3	14.0
P22/T7	16.3	16.2	32.6	9.9
ɸVPE25/T3	0.9	0.8	0.0	4.6
ɸVPE25/T7	1.1	1.1	0.0	3.3
T3/T7	1.3	1.3	2.1	0.7
T7/M13	465	748	4584	108
P22/M13	7587	12119	149630	1073
ɸVPE25/M13	530	807	61	356
T3/M13	587	956	9797	76

*In case of absence of purification biases, ratios of size normalized read abundances between phages would be identical in the unpurified control samples (MG) and the purified samples (FD, DTT and CsCl).

Differences in phage to phage ratios between the MG samples and the purified samples indicate that different methods discriminate against particular phages (Table 3, Additional file 1: Tables S2-S10). The most notable difference was a strong reduction of ɸVPE25 and T7 read abundances by the CsCl method. There are at least three potential explanations for this reduction. First, the ɸVPE25 and T7 particles may not have adequately accumulated in the density range extracted from the CsCl density gradient. Phage morphology is diverse and phages can vary widely in their buoyant densities, even when structurally related [23,31]. For example, the P22 and T7 phages both belong to the family *Podoviridae*, but have been shown to sediment at different density layers in a CsCl gradient [24]. Therefore, the bias against specific phage may have been introduced by extracting only one fraction from the CsCl gradient, however, the extracted density range was rather large, in theory, encompassing the densities of all phages in the sample. A second explanation for the observed reduction in read abundance in the CsCl samples is that some phage types degrade more rapidly in the CsCl gradient due to chemical or mechanical stresses. A third explanation would be that specific phage types rupture due to osmotic shock during buffer exchange releasing their genome. Osmotic shock is a common means to release nucleic acid from viruses [32]. Loss of nucleic acid during buffer exchange is unlikely, however, because the 50,000 MWCO ultrafiltration devices used are made to retain nucleic acids >300 bp. If osmotic rupturing had occurred, the genomic DNA of the ɸVPE25 and T7 bacteriophages (>38,000 bp) would have remained in the retentate used for nucleic acid extraction. Additionally, it was previously shown for the T7 phage that it is resistant to osmotic shock induced rupture [32]. One potential way to alleviate the biases introduced by the CsCl method would be to extract a larger density range from the CsCl gradient, which may lead to better retrieval of phages, but could also diminish the “cleansing” effect of the gradient by contamination carry over. Overall the biases introduced by the FD and DTT methods are less than in the CsCl method.

In conclusion, the high intra-method reproducibility of the FD and DTT methods allows quantitative cross-sample comparisons of phages. However, caution must be used when drawing conclusions about the abundances of individual phages within samples due to the fact that read abundance estimates within a sample can deviate by more than one order of magnitude from the actual input phage particle number (Table 3).

### Removal of bacterial and host DNA

All three purification methods were highly successful in removing both bacterial (*B. thetaiotaomicron* and *L. monocytogenes*) and mouse genomic DNA as compared to the MG samples (Figure 2 and Table 4). All three purification methods led to a >40,000-fold enrichment of phage reads to bacterial reads (Table 4). The greatest average fold change for phage to bacterial read enrichment was observed in the CsCl samples (49,077-fold) and the smallest in the FD samples (41,517-fold). These fold changes indicate a removal of >99.99% of the bacterial DNA by each purification method (Table 4). Host genomic DNA is a significant contaminant of fecal material and its presence should be considered when choosing an appropriate VLP purification method. For our study, the efficiency of removing mouse DNA from the samples differed greatly between the three purification methods. While the FD method only led to a 55-fold change in the ratio of phage reads to mouse reads indicating a removal of 98.1% of mouse DNA, the CsCl method led to a 768-fold enrichment indicating a removal of 99.87% of mouse DNA. The observed discrepancy in removal efficiency of bacterial DNA versus mouse DNA may be due to a larger fraction of mouse DNA existing as free DNA in mouse feces (i.e. not in nuclei or cells), which can easily pass through the filtration membrane, whereas the majority of the bacterial DNA is within cells that are efficiently removed by filtration.

**Table 4:** **Removal efficiency of mouse and bacterial DNA by different purification methods**

	**FD**	**DTT**	**CsCl**	**MG**
**Average % read abundance**				
Mouse	14.26	10.49	1.20	34.63
Phage^a^	82.57	86.79	96.13	3.62
Bacteria^b^	0.033	0.032	0.032	59.21
**Calculations based on average % read abundance**				
Phage/mouse ratio	5.79	8.27	80.31	0.10
Factor of ratio change compared to MG method	55	79	768	1
Estimated % decrease of read generating mouse DNA as compared to MG^c^	98.19	98.74	99.87	0.00
Phage/bacteria ratio	2537.74	2674.62	2999.83	0.06
Factor of ratio change compared to MG method	41518	43757	49078	1
Estimated % decrease of read generating bacterial DNA as compared to MG^c^	99.998	99.998	99.998	0.00

^a^:Sum of read abundances for all added bacteriophage (this excludes the phiX174 used as Illumina internal control); ^b^:Sum of read abundances for the two added bacteria – *L. monocytogenes* and *B. thetaiotaomicron*; ^c^:Calculated based on read abundances normalized to phage read abundance.

An alternative explanation for the higher amount of mouse DNA in the purified samples could be the introduction of mouse DNA contamination during sample processing. Such processing-contamination has, for example, been shown to occur when using certain types of DNA purification columns [33]. However, the observed differences in mouse DNA content between purification methods strongly suggest that mouse DNA is sample derived. Assuming that free mouse DNA is responsible for the presence of mouse reads in the purified metagenomes, this would suggest that free DNA was not completely removed by DNase digestion. There are two potential reasons for this. It is possible that specific regions of the mouse genome may have been protected from DNase digestion by adhering proteins protecting the DNA from degradation or that the conditions chosen for the DNase digestion were not sufficient to achieve a complete removal of mouse DNA. To check whether specific regions of the mouse genome were protected from DNase digestion we mapped the mouse reads to the mouse genome and visualized their location using the Integrative Genomics Viewer software (Vers. 2.3.34) [34]. We found that the reads were evenly distributed along the mouse genome suggesting that incomplete digestion of mouse DNA was not due to protection of specific genomic regions.

These data suggest that efforts to remove host DNA during the purification of VLPs from fecal samples need to be intensified. This could be achieved by increasing the DNase concentration during digestion. The DNase concentration that we used in this study was 10 U ml^−1^ and corresponds to what is recommended in a standard protocol for phage purification [25]; however, it has been suggested previously that in samples from animal hosts much higher DNase concentrations may be required to remove host DNA contamination [23]. Other laboratories have used higher DNase concentrations for sequencing analyses of viruses e.g. 100 U ml^−1^ for ocean virus metagenomes [16] and 500 U ml^−1^ for PCR based analyses of viruses in serum samples [35]. We would thus recommend using higher DNase concentrations for virus purification to achieve more efficient removal of host DNA. Additionally, in our study, DNase digestions were performed at room temperature. Digestion can also be done at 37°C and will yield accelerated degradation and greater removal of host DNA contamination. Finally, sequencing read data should be “decontaminated” using *in silico* methods by mapping the reads against a host reference genome to remove sequencing reads of any remaining host DNA.

### Phage genome recovery by genome assembly

To determine if whole phage genomes could be retrieved from the Illumina 100 bp paired-end read data, we performed de novo assemblies from all datasets (Table 5). We retrieved complete or almost complete genomes for four phages from all datasets (P22, ɸVPE25, T3 and T7, Table 5), despite the fact that we did not do any assembly procedure optimization (i.e. trying different parameters, binning reads or using assemblers optimized for metagenomes). Our assemblies even contained contigs of the ssDNA M13 phage for which only very few reads were sequenced. The largest M13 contig (~1 kbp) was assembled from the FD1 sample representing a significant portion of the 6.4 kbp genome of the M13 phage.

**Table 5:** **Assembly statistics**

	**Published sequence**	**FD1**	**FD2**	**DTT1**	**DTT2**	**CsCl1**	**CsCl2**	**MG1**	**MG2**
P22 No. of contigs	1	1	1	1	1	1	1	1	1
P22 largest contig (bp)^a^	41660	41737	41737	41737	41737	41737	41659	41737	41737
P22 coverage (x fold)	N/A	3651	3138	3925	4027	5489	4998	146	107
T7 No. of contigs	1	1	2	2	1	1	1	4	1
T7 largest contig (bp)	39937	39855	35301	35209	39344	39855	39855	35472	39855
T7 coverage (x fold)	N/A	256	220	257	273	168	202	16	11
T3 No. of contigs	1	1	4	4	2	1	1	2	1
T3 largest contig (bp)	38209	37540	33483	33392	37540	36121	37541	33656	36120
T3 coverage (x fold)	N/A	293	265	305	321	381	310	10	8
ɸVPE25 No. of contigs	1	1	1	1	1	1	1	1	2
ɸVPE25 largest contig (bp)	86524	86520	86522	86518	86542	86496	86505	86534	86540
ɸVPE25 coverage (x fold)	N/A	256	225	253	284	1.9	2.3	55	30
M13 No. of contigs	1	7	4	8	4	1	0	1	0
M13 largest contig (bp)	6407	1085	900	814	586	230	0	268	0
M13 coverage (x fold)	N/A	0.35	0.55	0.43	0.39	0.18	0	0.14	0

^a^:Assemblies of the P22 genome from our datasets are slightly larger than the reference genome, which is likely due to the quasi-circular nature of the P22 genome, which makes it impossible for the assembly algorithm to determine the exact start and end of the genome.

### Effects of nucleic acid composition of phage genomes

In our study we focused on the recovery of dsDNA phages. Nevertheless, we added two phages with non-dsDNA genomes (ɸ6 and M13) to the artificial microbiota sample to see if these phages could be recovered by any of the tested methods. To determine the recovery of the ɸ6 phage, which has a dsRNA genome, we did a cDNA synthesis using the FD and MG samples. The recovered ɸ6 RNA in these samples was too low and the sequencing library preparation failed. Consequently not a single read was retrieved for ɸ6 in any of the purified samples or in the MG samples. Surprisingly, for the M13 phage, which has a ssDNA genome, a small number of reads were sequenced in all purified samples and the MG samples. In theory sequencing of ssDNA should be prevented by the Illumina library preparation protocol which requires dsDNA as input [36]. T4 DNA ligase, commonly used for Illumina adapter ligation, works preferentially with dsDNA and excludes ssDNA. However, it has been shown that the T4 DNA ligase can ligate ssDNA albeit with a very low efficiency [37], which might explain why small amounts of M13 ssDNA were sequenced. This suggests that the tested methods are suitable for qualitatively assessing ssDNA viruses, if a large enough number of sequencing reads is generated. However, to get a clearer picture of RNA and ssDNA viruses additional steps should be added to these protocols.

Several approaches could be used to achieve greater sequencing coverage of ssDNA viruses. First, multiple displacement amplification (MDA), which is known to preferentially amplify ssDNA virusus, can be used to generate dsDNA [23,38-40]. MDA, however, has the caveat of introducing strong biases and the resulting sequences can only be used for a qualitative assessment of virus diversity and not quantitative analyses [19-21]. Second, random hexamer primers and DNA polymerase I can be used to convert ssDNA to dsDNA [41]. Third, ssDNA ligase can be used to ligate Illumina adapters directly to ssDNA during library preparation, however, this method has not been tested on viral DNA [30]. For the analysis of RNA viruses, RNA can be amplified and converted to dsDNA by reverse transcriptase [23,41]. However, care must be taken to avoid RNA degradation by nucleases during sample preparation. Furthermore, nucleic acid types can be separated using hydroxyapatite chromatography prior to dsDNA generation [41].

## Conclusions

In this study we used artificial microbiota samples, with known bacterial and viral composition, to evaluate four methods for the purification of VLPs from feces for quantitative comparisons of intestinal viromes. One method (PEG) failed during the purification procedure. All other methods succeeded in isolating VLPs and are suitable for quantitative sample comparison if one considers their limitations and pitfalls (discussed above). There are some notable differences in the ease of use, throughput, and performance of the different methods (Table 1). These differences should be considered when choosing the appropriate method. While the CsCl method outperformed the FD and DTT methods in removal efficiency of host derived DNA, the FD and DTT methods showed a lower discrimination against specific phage species and also yielded more total DNA. An additional consideration during study design should be the number of samples that will be analyzed. The FD and DTT methods allow for a much higher sample throughput, because they do not include the time consuming CsCl density gradient centrifugation step, which also limits the number of samples that can be processed in parallel (Table 1). As discussed above, the lower efficiency of host DNA removal in the FD and DTT methods may be alleviated by increasing DNase concentrations during DNase digestion. In this study we only observed small differences in the performance of the FD and DTT methods. Therefore, the DTT method can be applied to the processing of large scale fecal samples when mucus degradation is required to prevent filter clogging during VLP purification. A crucial additional consideration for the design of virome studies is the inclusion of appropriate negative controls to be carried from sample purification to metagenome sequencing and in silico analyses [42].

## Methods

### Mice

Germ-free C57BL/6 J mice were bred and reared in sterile isolators [43] at the UT-Southwestern Medical Center’s animal barrier facility. Animal protocols were approved by the Institutional Animal Care and Use Committees of UT-Southwestern.

### Bacteria, bacteriophages and culturing conditions

A total of six phages were used in this study. Three phages belonging to the Family *Podoviridae*, with linear dsDNA genomes, were purchased from the American Type Culture Collection (ATCC®, Manassas, VA). These phages included P22 (ATCC® 19585-B1™), T7 (ATCC® BAA-1025-B2™), and T3 (ATCC® BAA-1025-B1™), the latter was originally sold to us as T4 (ATCC® 11303-B4™). During the course of our study we determined that the T4 phage stock prepared by ATCC® was actually the T3 type strain. Phage ɸVPE25 was isolated from a municipal waste water source and the ɸVPE25 genome has been sequenced (manuscript in preparation). ɸVPE25 is a member of the *Siphoviridae* family of dsDNA viruses and infects *Enterococcus faecalis* (data not shown). In addition to dsDNA phages we used phages M13 (New England Biolabs, Ipswich, MA) which belongs to the family *Inoviridae* and contains a ssDNA genome and ɸ6 (a gift from P. Turner) a member of the *Cystoviridae* family harboring a segmented dsRNA genome [44].

All bacterial hosts were grown aerobically at 37°C except for *Pseudomonas syringae* pathovar *phaseolicola* HB10Y which was grown aerobically at 25°C [44]. *Escherichia coli* B (ATCC® 11303™) and *Salmonella enterica* subsp. *enterica* serovar *typhimurium* LT2 (ATCC® 19585™) were grown in ATCC® 129 medium (per liter; 3 g Beef Extract, 5 g Peptone, and 5 g NaCl), *E. faecalis* V583 was grown in Bacto® Brain Heart Infusion medium (BHI, Becton Dickinson, Franklin Lakes, NJ) [45], and *P. phaseolicola* was grown in LC medium (per liter; 10 g tryptone, 5 g yeast extract, and 10 g NaCl per liter, pH 7.5). For agar overlays during phage propagation, bacterial hosts were grown on their respective medium containing 1.5% base agar and 0.7% top agar. The bacteria used for the artificial intestinal microbiota were grown as follows; *Listeria monocytogenes* EGD-e [46] was grown aerobically on BHI and *Bacteroides thetaiotaomicron* VPI5482 [47] was grown anaerobically using the GasPak™ EZ Container System (Becton Dickinson) on TYG medium (per liter; 10 g tryptone, 5 g yeast extract, 2 g D-glucose, 0.5 g cysteine, 13.6 g KH_2_PO_4_, 17.4 g K_2_HPO_4_, 0.25 g NaHCO_3_, 0.4 g FeSO_4_, 80 mg NaCl, 20 mg MgSO_4_ · 7H_2_O, 8 mg CaCl_2_ · 2H_2_O, 1 g Vitamin-K, 0.25 g resazurin, 0.24 mg hematin [prepared by dissolving 12 mg of hematin in 10 ml of 0.2 M histidine, pH 8.0]).

### Phage propagation

- *P22, T7, and T3:* Propagation and purification of the enteric phages P22, T7, and T3 were similar. A single colony of *E. coli* B (T7 and T3) or *S. typhimurium* LT2 (P22) was inoculated into 5 ml of ATCC® 129 medium and grown overnight at 37°C with shaking (250 rpm). The bacteria were subcultured into 300 ml of fresh ATCC® 129 medium to an OD_600_ of 0.015. The cultures were grown for 2.5 hrs at 37°C with shaking. Lyophilized phages provided by ATCC® were reconstituted in 1 ml of ATCC® 129 medium and 30 μl of each phage solution was added to its respective host strain’s culture. These cultures were incubated for 3 hours (T7 and T3) and 3.5 hours (P22) at 37°C with shaking to achieve host lysis. The cultures were transferred to 500 ml centrifuge bottles and spun at 2820 × g for 20 min at 4°C. Any remaining bacterial pellet was discarded and the culture supernatants were filtered using a 0.22 μm bottle top filter (Corning, Tewksbury, MA). The clarified culture supernatants were treated with 10 U ml^−1^ each of RNase and DNase (Sigma-Aldrich, St. Louis, MO) for 1 hr at room temperature. 1 M solid NaCl and 10% (w/v) PEG 8000 was then added to the culture fluid and incubated on ice for four hours. The precipitated phage particles were transferred to a clean 500 ml centrifuge bottle and spun at 8000 × g for 20 min at 4°C. The phage pellets were resuspended in 3 ml of SM-plus buffer (100 mM NaCl, 50 mM Tris · HCl, 8 mM MgSO_4_ · 7H_2_O, 5 mM CaCl_2_ · 2H_2_O, pH 7.4). The resuspended phages were layered on top of CsCl step gradients consisting of increasing CsCl densities of 1.7 g/ml, 1.6 g/ml, and 1.42 g/ml (P22 and T3) and 1.7 g/ml, 1.6 g/ml, and 1.45 g/ml (T7) in Ultra-Clear™ 14 × 89 mm centrifuge tubes (Beckman Coulter, Indianapolis, IN), followed by spinning in a Beckman Coulter XE-90 Ultracentrifuge at 66,000 × g for 16 hours at 4°C, using a SW Ti-41 swinging bucket rotor. After ultracentrifugation phages were observed as visible hazy blue/white bands at the top of the 1.6 g/ml CsCl density zone. The phages within the condensed bands (0.5-1 ml) were removed from the gradient tubes using a syringe fitted with a 23 G needle and added to 5 ml of fresh SM-plus buffer. The samples were transferred to Amicon® Ultra Centrifugal Filters, 50,000 MWCO (EMD Millipore, Billerica, MA) and spun at 3220 × g for 5 min, resuspended in 4 ml of SM-plus buffer and centrifuged again. This step was repeated at least 3 times to remove the majority of the CsCl. After buffer exchange the final retentate was added to 2 ml of SM-plus buffer, filtered with a 0.22 μm SFCA syringe filter (Thermo Scientific-Nalgene, Waltham, MA), and stored at 4°C.
- *ɸ6:* For the amplification and purification of phage ɸ6 a method similar to Turner et al. was followed [44]. Briefly, a single colony of *P. phaseolicola* HB10Y was grown overnight in 5 ml of LC medium. 200 μl of the overnight *P. phaseolicola* culture was added to each of 6 sterile 14 ml round-bottom Falcon® tubes (Corning). To each tube 4.3×10^3^ PFU of ɸ6 was added and then mixed with 3 ml of molten LC top agar and poured onto the surface of an LC agar plate. The plates were incubated at room temperature overnight. 3 ml of LC broth was added to each plate and the top agar was collected. This mixture was centrifuged at 36,000 × g for 30 min at 4°C in an FX6100 fixed angle rotor (Beckman Coulter). The supernatant was transferred to a 25 × 89 mm polycarbonate cap assembly centrifuge tube (Beckman Coulter) and centrifuged at 73,000 × g for 2 hours at 4°C in a Ti 70 fixed angle rotor to pellet the phages. The phage pellet was resuspended in 1 ml of Buffer A (per liter; 1.9 g KH_2_PO_4_ · 3H_2_O, 0.25 g MgSO_4_ · 7H_2_O, pH 7.5). A sucrose density gradient was poured in an Ultra-Clear™ 14 × 89 mm centrifuge tube. The gradient steps consisted of 30%, 25%, 20%, 15%, and 10% sucrose dissolved in Buffer A. The suspended phages were layered on top of the 10% sucrose step and centrifuged in a SW Ti-41 rotor at 66,000 × g for 1 hr at 15°C. The phage band was removed from the gradient (~1 ml) using a syringe and 23 G needle and suspended in Buffer A. The sample was transferred to a 25 × 89 mm polycarbonate cap assembly centrifuge tube and centrifuged at 73,000 × g for 2 hrs. The resulting supernatant was saved and the phage pellet resuspended in 0.6 ml of Buffer A. The supernatant was centrifuged a second time to collect any remaining phages for 4 hrs at 73,000 × g. Again the supernatant was saved and the pellet was suspended in 0.6 ml of Buffer A. It was determined by plaque assay that the recovered phages from the pellet were of insufficient titer and upon analysis of the supernatants, a large proportion of the phages did not pellet by ultracentrifugation. Therefore, the pelleted phage samples and the decanted supernatants were pooled and transferred to an Amicon® Ultra Centrifugal Filter, 10,000 MWCO (EMD Millipore) and spun at 3220 × g for 5 min. The filtration unit was filled with fresh Buffer A and spun at 3220 × g for 7 min. This was repeated a second time resulting in a final retentate volume of 1.5 ml which was stored at 4°C.
- *ɸVPE25*: A 5 ml culture of BHI was inoculated with a single colony of *E. faecalis* V583 and grown overnight. The next day 300 ml of BHI medium was inoculated with the overnight culture to an OD_600_ of 0.025. The culture was transferred to a 37°C shaking incubator and grown until the OD_600_ reached 0.7. The culture was removed from the incubator and 10 mM MgSO_4_ · 7H_2_O was added followed by the addition of ɸVPE25 particles. The culture was incubated at room temperature for 10 min and then placed in the 37°C shaking incubator for 4 hours. The culture was transferred to a 500 ml centrifuge bottle and spun at 2820 × g for 20 min at 4°C. The supernatant was collected and filtered through a 0.45 μm bottle top filter (Thermo Scientific-Nalgene). The filtered culture fluid was treated with 10 U ml^−1^of both RNase and DNase for 1 hr at room temperature. 1 M solid NaCl and 10% (w/v) polyethylene glycol (PEG) 8000 was dissolved into the culture fluid and incubated on ice overnight at 4°C. The phages were pelleted by centrifugation at 8000 × g for 20 min at 4°C. The phage pellet was resuspended in 3 ml of SM-plus buffer and extracted with 1/5^th^ volume of chloroform and centrifuged at 16,000 × g for 2 min. The aqueous phase containing the phages was collected, brought up to 4.5 ml with SM-plus buffer. 2.2 g of CsCl was dissolved into the sample, which was placed on top of the 1.45 g/ml CsCl density layer of a CsCl gradient consisting of 1.7 g/ml, 1.5 g/ml, and 1.45 g/ml steps. This was placed in a SW Ti-41 rotor and spun at 59,764 × g for 2 hrs at 4°C. The phage band (~1-2 ml) was removed using a syringe and 23 G needle and transferred to Slide-A-Lyzer® dialysis cassette, 10,000 MWCO, (Thermo Scientific) and dialyzed twice against 2 L of SM-plus buffer to remove the CsCl. After dialysis the phage sample was removed from the cassette and stored at 4°C.

Phage titers were determined with the standard soft-agar overlay method using the phage specific host strains and media [25,48].

### Quantification of phage particles by fluorescence microscopy

To determine the absolute quantities of dsDNA phages P22, T3, T7, and ɸVPE25 in stock solutions, a method similar to that described by Thurber et al. was used [23]. Concentrated phage stocks were diluted 10,000X-100,000X in SM-plus buffer. An aliquot of diluted phage was added to 5 ml of SM-plus and applied to the column of a 25-mm microanalysis filter holder with a fritted glass support (VWR International, Radnor, PA) holding a 25-mm 0.02 μm aluminum oxide Annodisc filter (GE Healthcare Bio-Sciences, Pittsburgh, PA). Vacuum was applied (<10 p.s.i.) until all of the fluid passed through the filter. Filtration was performed in triplicate. After filtration the filter was placed (sample side up) onto a 120 μl drop of 5X SYBR® Gold in a sterile petri dish and incubated at room temperature for 30 minutes in the dark. The filter was washed once by placing onto a 120 μl drop of SM-plus for 30 seconds and excess liquid was wicked away using a Kimwipe. Filters were mounted onto glass microscope slides with cover slips using Tris-buffered Fluoro-Gel (Electron Microscopy Sciences, Hatfield, PA) and phage particles were imaged on an Axio Imager.M1 fluorescence microscope (Carl Zeiss Microscopy, Göttingen, Germany) coupled to an X-Cite Series 120 illumination lamp (EXFO Life Sciences, Montreal, Quebec, Canada) at 1000X magnification. Fluorescent phage particles were counted from a total of 5 random fields per filter and averaged. Absolute phage numbers were calculated based on the average phage counts per field taking into account the area of one field (0.00596 mm^2^), the area of the Annodisc filter (490.874 mm^2^), and back calculating based on the dilution factor.

### Generation of the artificial microbiota master mix sample

One artificial gut microbiome sample master mix (for a total of 10 samples) was produced by the introduction of various pure cultures of bacteria and phages into feces from germ-free C57BL/6 J mice. 2.5×10^9^ CFU each of *L. monocytogenes* and *B. thetaiotaomicron* were added to 2.7 g of freshly collected germ-free mouse feces (0.27 g feces per sample). Then, for each of the six phages – P22, T7, T3, ɸVPE25, M13, and ɸ6 – 8.3×10^8^ PFU each were added to the sample resulting in a total of 5×10^9^ phage particles in the master mix. The number of phage particles to be used in the master mix was determined in a pre-experiment by quantifying the amount of DNA in 10^10^ PFU of ɸVPE25, which was 2,355 ng, and the amount of DNA that could be extracted from VLPs isolated by CsCl density gradient centrifugation from 0.6 g of conventional mouse feces, which was 12 ng. Based on this we estimated that ~5×10^7^ VLPs could be isolated from 0.6 g of mouse feces. To account for VLP loss during purification procedures we added 10-fold excess PFU of each phage for each of the 10 individual samples in the master mix. After addition of the bacteria and phages to the feces master mix, 12 ml of SM-plus buffer was added and the master mix was homogenized by rotor and stator (Omni International, Kennesaw, GA). The master mix was then weighed and aliquoted into 10 equal samples.

### Purification procedures

Immediately upon allocation of the 10 individual samples, two samples were extracted for nucleic acid (see below). These samples denote the *Complete Metagenome (MG)*. The remaining 8 samples were centrifuged at 2500 × g for 5 min, the supernatant was collected and then centrifuged a second time at 5000 × g for 15 min. At this point various modifications to the procedure were preformed to enrich for virus-like particles (VLPs) by different means: *Purification by filtration (FD)* – Two samples were filtered through a 0.45 μm Millex®-HV low protein binding PVDF syringe filter (EMD Millipore) which was washed with 500 μl of SM-plus buffer. The filtered fluid was treated with 10 U ml^−1^ of DNase at room temperature for 1 hour and then extracted for nucleic acids. *Mucolytic agent and filter purification (DTT)* – One problem that occurs when using large quantities of intestinal contents for phage isolation is interference of intestinal mucus during filtration. A procedure that can be used to reduce the viscosity of mucous containing samples during phage preparation, prior to filtration, is treatment with the reducing reagent dithiothreitol (DTT) [13]. However, the effects of DTT on phage particle recovery during purification are not well understood. Therefore, two samples were first treated with 6.5 mM DTT for 1 hr at 37°C, filtered through a 0.45 μm PVDF syringe filter which was washed with 500 μl of SM-plus buffer, treated with 10 U ml^−1^ of DNase at room temperature for 1 hour, and the nucleic acid extracted. *Purification by filtration and cesium chloride density gradient centrifugation (CsCl)* – A common procedure for the purification of phages from complex intestinal contents is to purify the particles by CsCl centrifugation [7,8,23]. Two samples were filtered through a 0.45 μm syringe filter, washed with 500 μl of SM-plus buffer, and treated with 10 U ml^−1^ of DNase at room temperature for 1 hour. After DNase treatment the samples were loaded onto a CsCl gradient composed of 1.7 g/ml, 1.5 g/ml, 1.35 g/ml steps and centrifuged for 16 hours in a SW Ti-41 rotor at 4°C. The interface between the 1.35 and 1.5 g/ml density region (~1 ml) was collected and the CsCl was removed by three 4 ml volume SM-plus buffer exchanges in an Amicon® Ultra Centrifugal Filter (50,000 MWCO). The samples were then extracted for total DNA. *Purification by filtration and precipitation (PEG)* – The final method was based on a phage precipitation technique where PEG8000 is added to sequester water molecules forcing the phages to aggregate. The precipitated phages can then be collected by centrifugation [24,25]. Two samples were filtered through a 0.45 μm PVDF syringe filter which was washed with 500 μl of SM-plus buffer, treated with 10 U ml^−1^ of DNase at room temperature for 1 hour. To each sample 1 M NaCl and 10% (w/v) PEG8000 was added, the phages were allowed to precipitate on ice for 2 hours. The precipitate was collected by centrifugation at 8000 × g for 20 minutes and resuspended in 1 ml of SM-plus buffer. To remove the PEG8000 prior to nucleic acid extraction a buffer exchange was attempted by the addition of 4 ml of SM-plus to the sample and centrifugation in a Amicon® Ultra Centrifugal Filter (50,000 MWCO). However, the PEG precipitation resulted in an extremely viscous solution that could not pass through the centrifugal filter during buffer exchange. Therefore, this sample was omitted from further analysis.

An important consideration for the purification method design is the choice of pore size to be used for filtration steps. The right balance between removing the unwanted microbial cells and letting viruses pass has to be found. Commonly used pore sizes for virus isolation are 0.2 and 0.45 μm [16,49-51]. We chose a 0.45 μm pore size for the tested purification methods because several bacteriophage groups from the mammalian intestine have members that are larger than 0.2 μm (e.g. Myoviridae and Siphoviridae [52]). Additionally, giant eukaryotic viruses (~400 nm) were recently discovered in human intestinal content [50]. Since, most microbial cells in the mammalian intestine are larger than 0.45 μm [53], use of 0.45 μm filters should be possible. In contrast, some environments harbor abundant bacteria smaller than 0.45 μm, for example *Pelagibacter ubique* in the open ocean [54], where the use of a 0.2 μm pore size for virus isolation may be crucial.

### Nucleic acid extraction

For all samples, total nucleic acids were extracted using the following protocol. 50 μg/ml Proteinase-K and 0.5% sodium dodecyl sulfate (SDS) was added to each sample and incubated at 56°C for 1 hour. Samples were mixed with an equal volume of phenol/chloroform/isoamyl alcohol and vigorously extracted by shaking for 10 sec. The samples were centrifuged at 16,000 × g for 2 min. The aqueous phase was transferred to a clean microfuge tube and extracted with an equal volume of chloroform. The samples were centrifuged at 16,000 × g for 2 min and the chloroform extraction was repeated. The aqueous phase was transferred to a clean microfuge tube and the nucleic acids precipitated by the addition of 0.3 M sodium acetate (pH, 7.0) and 2.5 volumes of isopropanol. The samples were incubated at −20°C for 2 hours and then centrifuged at 16,000 × g for 30 minutes. The precipitated nucleic acid was washed once with 700 μl of 70% ethanol and centrifuged at 16,000 × g for 10 minutes. The supernatant was decanted and the pellets were allowed to dry at room temperature for 10 min. The nucleic acid was resuspended in EB buffer (10 mM Tris · HCl, pH 8.5) and further purified using the MinElute® Reaction Cleanup Kit (Qiagen, Valencia, CA).

### cDNA synthesis

For the preparation of cDNA from total RNA, nucleic acid extracted metagenome samples were treated with 10 U ml^−1^ of DNase for 1 hour at room temperature. RNA was purified using an RNeasy Mini Kit (Qiagen). RNA was eluted from the RNeasy columns in 30 μl of water. 5 μM of random hexamer oligonucleotides (Life Technologies, Carlsbad, CA) was added to 10 μl of the eluate and incubated for 5 minutes at 65°C. 5 μl of the RNA-random hexamer mix was added to 200 U of M-MLV reverse transcriptase (Life Technologies) and cDNA was synthesized using the cycling parameters, 25°C for 10 min, 42°C for 1 hr. cDNA was subsequently purified using the MinElute® Reaction Cleanup Kit. cDNA systhesis yielded a DNA concentration below the limit of detection (<0.2 ng/μl as determined by Qubit (Life Technologies) analysis). These samples were of insufficient quantity to generate a quality library preparation as determined by a Bioanalyzer 2100 (Agilent Technologies, Santa Clara, CA).

### Preparation of sequencing libraries and sequencing on Illumina HiSEq 2500

DNA concentration was determined using the Qubit dsDNA HS kit. DNA quantity and quality was further assessed by product size and concentration using a Bioanalyzer 2100. DNA was sheared with an S2 focused-ultrasonicator (Covaris, Woburn, MA) to achieve a target size range of fragments of 100–900 bp. The KAPA High Throughput Library Preparation Kit with Standard PCR Library Amplification (KAPA Biosystems, Wilmington, MA) was used to generate Illumina sequencing libraries according to the manufacturer’s instructions. Illumina TruSeq adapters with 6 bp indices were used to enable multiplexed sequencing. Indices on adapters were chosen such as to achieve highest discriminating power between individual libraries. All libraries were amplified with seven PCR cycles. This cycle number was chosen based on the minimal number of PCR cycles necessary for the sample with lowest input DNA (CsCl2) and used for all samples to avoid differences between samples due to varying cycle numbers. Illumina libraries were subjected to dual size selection using AMPure XP beads (Beckman Coulter) with a target size of 300 to 1000 bp. Library quality was measured on a Bioanalyzer 2100 by product size and concentration. All eight samples were sequenced on a single lane on an Illumina HiSeq 2500 (Illumina, San Diego, CA) in paired-end mode. About 14 million 100 bp paired-end reads were generated for each sample. Sequencing reads were demultiplexed based on the 6 bp index integrated in the Illumina TruSeq adapter sequences allowing for one mismatch using the CASAVA software provided by Illumina.

### Read mapping and quantification

Raw reads were mapped against a set of reference genomes to quantify reads per organism. The following reference genomes were used (NCBI accession numbers if not otherwise noted): bacteriophages, M13 (JX412914.1), P22 (AF217253.1 and AB426868.1), ɸ6 (M17461.1, M17462.1 and M12921.1), T3 (KC960671.1), T7 (NC_001604.1) and ɸVPE25 (unpublished, available upon request); bacteria, *L. monocytogenes* EGD-e (AL591824.1), *B. thetaiotaomicron* VPI-5482 (AE015928.1 and AY171301.1), *E. coli* BL21(DE3) (NC_012971.2), *S. enterica* subsp. *enterica* serovar Typhimurium str. LT2 (NC_003197.1 and NC_003277.1), *P. syringae* pv. *phaseolicola* 1448A (CP000058.1, CP000059.1 and CP000060.1) and *Enterococcus faecalis* V583 (NC_004668.1, NC_004669.1, NC_004671.1 and NC_004670.1); phiX174 used as an internal Illumina control (J02482.1); the mouse reference genome (mm10) and the human reference genome (hg38) were downloaded through the UCSC Genome Browser [55].

Reads were mapped onto all reference genomes in parallel using the BBSplit tool, which is part of the BBMap short read aligner tool set (Version 32.15) [56]. The ‘ambig2’ parameter was set to ‘split’ , resulting in reads that map to more than one reference genome being written into separate files for ambiguous reads. Read mapping statistics for each reference genome were generated by setting the ‘refstats’ parameter. Additionally the following default parameters were used by the program: match = long, fastareadlen = 500, minapproxhits = 2, minratio = 0.9, maxindel = 20, trim = both, untrim = true. Reads that did not map to any of the references were output into separate FASTQ files with unmapped reads.

### Mouse read mapping visualization

To visualize the distribution of reads mapping to the mouse reference genome, BAM read alignment files were generated using BBMap. BAM files plus the mouse reference genome were then loaded into the Integrative Genomics Viewer (Version 2.3.34) [34,57] and read mapping along all chromosomes was inspected visually.

### Trimming, assembly and extraction of phage contigs

To determine how well phage genomes could be reconstructed from the metagenomes, we assembled the metagenomes and then checked for the number and size of phage contigs. We trimmed the raw reads using the ‘nesoni clip’ tool from the Nesoni high-throughput sequencing data analysis toolset (Version 0.114, http://www.vicbioinformatics.com/software.nesoni.shtml) for Illumina adapters and a minimum quality of 2. Additionally, we removed the first 9 and last 5 bases from each read. Read quality statistics were checked before and after trimming with the FastQC tool (Version 0.10.1) [58]. Trimmed reads were error corrected with the BayesHammer tool [59] integrated in the SPAdes pipeline and then assembled using the SPAdes assembler (Version 3.0.0) [60,61] in multi-cell mode using k-mer lengths of 21, 33, 55 and 77. We searched the resulting assemblies for phage contigs by querying the assemblies with the phage reference genomes using BLASTN (Version 2.2.29+) [62,63].

### Availability of supporting data

The data sets supporting the results of this article are available in the European Nucleotide Archive, PRJEB6941, http://www.ebi.ac.uk/ena/data/view/PRJEB6941.

## Additional file

Additional file 1:
**Read mapping details and phage enumeration comparison.** **Table S1.** Percent unambiguously mapped reads. **Table S2.** Percent unambiguously mapped reads normalized to genome size. **Table S3.** Details on read mapping for sample FD1. **Table S4.** Details on read mapping for sample FD2. **Table S5.** Details on read mapping for sample DTT1. **Table S6.** Details on read mapping for sample DTT2. **Table S7.** Details on read mapping for sample CsCl1. **Table S8.** Details on read mapping for sample CsCl2. **Table S9.** Details on read mapping for sample MG1. **Table S10.** Details on read mapping for sample MG2. **Table S11.** Plaque forming unit (PFU) count versus virus-like particle (VLP) count for dsDNA phages in phage stocks used to prepare the artificial microbiome sample.

## Abbreviations

CFU: Colony forming unit
dsDNA: Double-stranded DNA
ssDNA: Single-stranded DNA
dsRNA: Double-stranded RNA
MDA: Multiple displacement amplification
PFU: Plaque forming unit
VLP: Virus-like particle
