# Supplementary material for: Evaluation of methods to purify virus-like particles for metagenomic sequencing of intestinal viromes
Source: BMC Genomics. 2015 Jan 22;16(1):7. doi: 10.1186/s12864-014-1207-4 (PMC4308010; doi:10.1186/s12864-014-1207-4)
Supplement: Additional file 1: — Read mapping details and phage enumeration comparison. Table S1. Percent unambiguously mapped reads. Table S2. Percent unambiguously mapped reads normalized to genome size. Table S3. Details on read mapping for sample FD1. Table S4. Details on read mapping for sample FD2. Table S5. Details on read mapping for sample DTT1. Table S6. Details on read mapping for sample DTT2. Table S7. Details on read mapping for sample CsCl1. Table S8. Details on read mapping for sample CsCl2. Table S9. Details on read mapping for sample MG1. Table S10. Details on read mapping for sample MG2. Table S11. Plaque forming unit (PFU) count versus virus-like particle (VLP) count for dsDNA phages in phage stocks used to prepare the artificial microbiome sample. [file 12864_2014_1207_MOESM1_ESM.pdf]

Table S1. % Unambiguously mapped reads

Organism	***Genome size (kbp)***	***Samples***							
		**FD1**	**FD2**	**DTT1**	**DTT2**	**CsCl1**	**CsCl2**	**MG1**	**MG2**
B. thetaiotaomicron VPI5482	6383	0.035	0.027	0.027	0.035	0.026	0.035	61.070	51.498
E. coli B	4624	0.001	0.001	0.001	0.001	0.001	0.002	0.001	0.001
E. faecalis V583	3408	6 E-5	3 E-5	6 E-5	1 E-5	1 E-5	2 E-4	1 E-5	0.000
Human	3209286	0.029	0.023	0.014	0.019	0.032	0.009	0.016	0.014
L. monocytogenes EGD-e	2986	0.002	0.001	0.001	0.002	0.001	0.002	3.153	2.706
M13	6	0.001	0.001	0.001	0.001	1 E-4	6 E-5	3 E-4	2 E-4
Mouse	2780048	15.581	12.938	10.303	10.682	0.961	1.433	28.444	40.806
P22	42	63.821	66.215	69.108	68.103	88.684	87.821	2.476	1.432
ϕVPE25	85	8.920	9.476	8.871	9.635	0.063	0.085	1.888	0.810
ϕX174	5	0.417	0.314	0.316	0.456	0.396	0.484	0.414	0.344
P. phaseolicola HB10Y	6200	3 E-5	2 E-5	4 E-5	1 E-5	3 E-5	1 E-5	1 E-5	1 E-5
S. typhimurium LT2	5022	1.167	1.217	1.251	1.273	1.465	1.606	0.043	0.024
T3	38	4.573	4.535	4.908	4.884	5.565	4.892	0.159	0.094
T7	40	3.847	3.741	4.029	4.043	2.142	3.008	0.248	0.132
Unmapped or ambiguous	N/A	1.605	1.510	1.169	0.867	0.663	0.624	2.089	2.140
**% Sum of unambiguously mapped reads**		**98.39**	**98.49**	**98.83**	**99.13**	**99.34**	**99.38**	**97.91**	**97.86**

Table S2. % Unambiguously mapped reads normalized to genome size

Organism	***Genome size (kbp)***	***Samples***							
		**FD1**	**FD2**	**DTT1**	**DTT2**	**CsCl1**	**CsCl2**	**MG1**	**MG2**
B. thetaiotaomicron VPI5482	6383	2 E-4	2 E-4	2 E-4	3 E-4	1 E-4	2 E-4	5.172	6.344
E. coli B	4624	2 E-5	1 E-5	1 E-5	1 E-5	1 E-5	1 E-5	1 E-4	1 E-4
E. faecalis V583	3408	9 E-7	5 E-7	8 E-7	2 E-7	8 E-8	2 E-6	1 E-6	0.000
Human	3209286	5 E-7	4 E-7	2 E-7	3 E-7	4 E-7	1 E-7	3 E-6	3 E-6
L. monocytogenes EGD-e	2986	3 E-5	2 E-5	2 E-5	3 E-5	1 E-5	2 E-5	0.571	0.713
M13	6	0.009	0.012	0.009	0.004	0.001	4 E-4	0.023	0.032
Mouse	2780048	3 E-4	2 E-4	2 E-4	2 E-4	1 E-5	2 E-5	0.006	0.012
P22	42	78.945	80.265	80.524	78.866	88.280	87.380	31.874	26.802
ϕVPE25	85	5.452	5.676	5.107	5.513	0.031	0.042	12.007	7.491
ϕX174	5	4.333	3.197	3.096	4.437	3.314	4.042	44.730	54.066
P. phaseolicola HB10Y	6200	2 E-7	1 E-7	3 E-7	5 E-8	2 E-7	5 E-8	6 E-7	7 E-7
S. typhimurium LT2	5022	0.012	0.012	0.012	0.012	0.012	0.013	0.005	0.004
T3	38	6.252	6.076	6.321	6.251	6.123	5.380	2.259	1.942
T7	40	4.997	4.762	4.929	4.916	2.239	3.143	3.352	2.596

Table S3. Details on read mapping, sample FD1

Organism	***% Unambiguous reads***	***Unambiguous MB***	***% Ambiguous reads***	***Ambiguous MB***	***Unambiguous reads***	***Ambiguous reads***	***Genome size (kbp)***	***Unambigious reads/genome size***	***% Normmalized to genome size***
B. thetaiotaomicron VPI5482	0.035	0.988	0.001	0.035	9880	346	6383	1.548	2 E-4
E. coli B	0.001	0.039	0.254	7.133	392	71332	4624	0.085	2 E-5
E. faecalis V583	6 E-5	0.002	1 E-5	4 E-4	16	4	3408	0.005	9 E-7
Human	0.029	0.820	0.004	0.107	8196	1068	3209286	0.003	5 E-7
L. monocytogenes EGD-e	0.002	0.054	1 E-4	0.003	536	28	2986	0.180	3 E-5
M13	0.001	0.029	5 E-5	0.001	292	14	6	48.667	0.009
Mouse	15.581	438.368	0.342	9.620	4383684	96198	2780048	1.577	3 E-4
P22	63.821	1795.540	0.575	16.165	17955402	161654	42	427509.571	78.945
ϕVPE25	8.920	250.943	0.238	6.692	2509432	66924	85	29522.729	5.452
ϕX174	0.417	11.734	7 E-5	0.002	117336	20	5	23467.200	4.333
P. phaseolicola HB10Y	3 E-5	0.001	0	0	8	0	6200	0.001	2 E-7
S. typhimurium LT2	1.167	32.838	0.059	1.648	328380	16476	5022	65.388	0.012
T3	4.573	128.650	0.107	2.998	1286500	29982	38	33855.263	6.252
T7	3.847	108.234	0.346	9.731	1082336	97306	40	27058.400	4.997

Table S4. Details on read mapping, sample FD2

Organism	***% Unambiguous reads***	***Unambiguous MB***	***% Ambiguous reads***	***Ambiguous MB***	***Unambiguous reads***	***Ambiguous reads***	***Genome size (kbp)***	***Unambigious reads/genome size***	***% Normmalized to genome size***
B. thetaiotaomicron VPI5482	0.027	0.629	0.001	0.022	6290	224	6383	0.985	2 E-4
E. coli B	0.001	0.026	0.242	5.651	258	56508	4624	0.056	1 E-5
E. faecalis V583	3 E-5	0.001	3 E-5	0.001	8	6	3408	0.002	5 E-7
Human	0.023	0.533	0.003	0.067	5332	668	3209286	0.002	4 E-7
L. monocytogenes EGD-e	0.001	0.025	8 E-5	0.002	254	18	2986	0.085	2 E-5
M13	0.001	0.033	6 E-5	0.001	330	14	6	55.000	0.012
Mouse	12.938	301.963	0.256	5.965	3019630	59646	2780048	1.086	0.0002
P22	66.215	1545.432	0.477	11.125	15454324	111252	42	367960.095	80.265
ϕVPE25	9.476	221.175	0.218	5.080	2211748	50798	85	26020.565	5.676
ϕX174	0.314	7.328	7 E-5	0.002	73276	16	5	14655.200	3.197
P. phaseolicola HB10Y	2 E-5	0.0004	0	0	4	0	6200	0.001	1 E-7
S. typhimurium LT2	1.217	28.394	0.051	1.197	283940	11968	5022	56.539	0.012
T3	4.535	105.851	0.088	2.056	1058512	20560	38	27855.579	6.076
T7	3.741	87.314	0.317	7.406	873140	74062	40	21828.500	4.762

Table S5. Details on read mapping, sample DTT1

Organism	***% Unambiguous reads***	***Unambiguous MB***	***% Ambiguous reads***	***Ambiguous MB***	***Unambiguous reads***	***Ambiguous reads***	***Genome size (kbp)***	***Unambigious reads/genome size***	***% Normmalized to genome size***
B. thetaiotaomicron VPI5482	0.027	0.758	0.001	0.032	7580	318	6383	1.188	2 E-4
E. coli B	0.001	0.033	0.269	7.519	332	75188	4624	0.072	1 E-5
E. faecalis V583	0.000	0.002	0	0.000	16	0	3408	0.005	8 E-07
Human	0.014	0.396	0.002	0.056	3956	556	3209286	0.001	2 E-7
L. monocytogenes EGD-e	0.001	0.035	4 E-5	0.001	348	10	2986	0.117	2 E-5
M13	0.001	0.031	1 E-4	0.003	312	32	6	52.000	0.009
Mouse	10.303	288.510	0.176	4.916	2885104	49160	2780048	1.038	2 E-4
P22	69.108	1935.189	0.424	11.860	19351886	118598	42	460759.190	80.524
ϕVPE25	8.871	248.400	0.185	5.188	2483996	51880	85	29223.482	5.107
ϕX174	0.316	8.859	9 E-5	0.002	88588	24	5	17717.600	3.096
P. phaseolicola HB10Y	4 E-5	0.001	0	0.000	10	0	6200	0.002	3 E-7
S. typhimurium LT2	1.251	35.026	0.056	1.571	350264	15714	5022	69.746	0.012
T3	4.908	137.444	0.094	2.645	1374444	26450	38	36169.579	6.321
T7	4.029	112.825	0.347	9.725	1128254	97248	40	28206.350	4.929

Table S6. Details on read mapping, sample DTT2

Organism	***% Unambiguous reads***	***Unambiguous MB***	***% Ambiguous reads***	***Ambiguous MB***	***Unambiguous reads***	***Ambiguous reads***	***Genome size (kbp)***	***Unambigious reads/genome size***	***% Normmalized to genome size***
B. thetaiotaomicron VPI5482	0.035	1.020	0.001	0.027	10200	274	6383	1.598	3 E-4
E. coli B	0.001	0.040	0.265	7.716	398	77156	4624	0.086	1 E-5
E. faecalis V583	1 E-5	4 E-4	0	0.000	4	0	3408	0.001	2 E-7
Human	0.019	0.546	0.002	0.054	5460	542	3209286	0.002	3 E-7
L. monocytogenes EGD-e	0.002	0.046	3 E-5	0.001	456	10	2986	0.153	3 E-5
M13	0.001	0.015	5 E-5	0.001	146	14	6	24.333	0.004
Mouse	10.682	311.100	0.094	2.748	3111004	27482	2780048	1.119	2 E-4
P22	68.103	1983.514	0.215	6.263	19835136	62634	42	472265.143	78.866
ϕVPE25	9.635	280.627	0.088	2.568	2806270	25680	85	33014.941	5.513
ϕX174	0.456	13.284	1 E-4	0.003	132840	34	5	26568.000	4.437
P. phaseolicola HB10Y	1 E-5	2 E-4	0	0	2	0	6200	3 E-4	5 E-8
S. typhimurium LT2	1.273	37.069	0.037	1.072	370688	10724	5022	73.813	0.012
T3	4.884	142.239	0.051	1.494	1422394	14938	38	37431.421	6.251
T7	4.043	117.759	0.308	8.984	1177588	89836	40	29439.700	4.916

Table S7. Details on read mapping, sample CsCl1

Organism	***% Unambiguous reads***	***Unambiguous MB***	***% Ambiguous reads***	***Ambiguous MB***	***Unambiguous reads***	***Ambiguous reads***	***Genome size (kbp)***	***Unambigious reads/genome size***	***% Normmalized to genome size***
B. thetaiotaomicron VPI5482	0.026	0.808	0.001	0.026	8084	264	6383	1.266	1 E-4
E. coli B	0.001	0.035	0.142	4.364	354	43640	4624	0.077	1 E-5
E. faecalis V583	1 E-5	2 E-4	0	0.001	2	8	3408	0.001	8 E-8
Human	0.032	0.974	0.001	0.029	9742	294	3209286	0.003	4 E-7
L. monocytogenes EGD-e	0.001	0.032	5 E-5	0.001	322	14	2986	0.108	1 E-5
M13	1 E-4	0.003	0	0.000	32	0	6	5.333	0.001
Mouse	0.961	29.432	0.026	0.791	294316	7912	2780048	0.106	1 E-5
P22	88.684	2716.358	0.204	6.252	27163578	62516	42	646751.857	88.280
ϕVPE25	0.063	1.935	0.002	0.051	19348	506	85	227.624	0.031
ϕX174	0.396	12.140	9 E-5	0.003	121400	28	5	24280.000	3.314
P. phaseolicola HB10Y	3 E-5	0.001	0	0	10	0	6200	0.002	2 E-7
S. typhimurium LT2	1.465	44.880	0.067	2.062	448800	20622	5022	89.367	0.012
T3	5.565	170.446	0.101	3.107	1704462	31074	38	44854.263	6.123
T7	2.142	65.608	0.191	5.848	656082	58476	40	16402.050	2.239

Table S8. Details on read mapping, sample CsCl2

Organism	***% Unambiguous reads***	***Unambiguous MB***	***% Ambiguous reads***	***Ambiguous MB***	***Unambiguous reads***	***Ambiguous reads***	***Genome size (kbp)***	***Unambigious reads/genome size***	***% Normmalized to genome size***
B. thetaiotaomicron VPI5482	0.035	0.984	0.001	0.031	9844	312	6383	1.542	2 E-4
E. coli B	0.002	0.055	0.201	5.658	546	56582	4624	0.118	1 E-5
E. faecalis V583	2 E-4	0.005	0	0.001	46	6	3408	0.013	2 E-6
Human	0.009	0.252	4 E-4	0.011	2524	110	3209286	0.001	1 E-7
L. monocytogenes EGD-e	0.002	0.046	1 E-4	0.003	458	30	2986	0.153	2 E-5
M13	6 E-5	0.002	0	0	18	0	6	3.000	4 E-4
Mouse	1.433	40.283	0.031	0.871	402832	8714	2780048	0.145	2 E-5
P22	87.821	2468.759	0.198	5.553	24687594	55526	42	587799.857	87.380
ϕVPE25	0.085	2.378	0.002	0.053	23782	530	85	279.788	0.042
ϕX174	0.484	13.594	1 E-4	0.003	135944	30	5	27188.800	4.042
P. phaseolicola HB10Y	1 E-5	2 E-4	0	0	2	0	6200	0.000	5 E-8
S. typhimurium LT2	1.606	45.159	0.065	1.830	451588	18302	5022	89.922	0.013
T3	4.892	137.518	0.084	2.354	1375184	23540	38	36189.053	5.380
T7	3.008	84.571	0.257	7.224	845710	72244	40	21142.750	3.143

Table S9. Details on read mapping, sample MG1

Organism	*% Unambiguous reads*	*Unambiguous MB*	*% Ambiguous reads*	*Ambiguous MB*	*Unambiguous reads*	*Ambiguous reads*	*Genome size (kbp)*	*Unambigious reads/genome size*	*% Normmalized to genome size*
B. thetaiotaomicron VPI5482	61.070	1810.581	0.558	16.549	18105810	165492	6383	2836.567	5.172
E. coli B	0.001	0.029	0.016	0.484	290	4844	4624	0.063	1 E-4
E. faecalis V583	1 E-5	2 E-4	0	0.005	2	52	3408	0.001	1 E-6
Human	0.016	0.467	0.007	0.216	4672	2156	3209286	0.001	3 E-6
L. monocytogenes EGD-e	3.153	93.478	0.062	1.848	934776	18484	2986	313.053	0.571
M13	3 E-4	0.008	0	0	76	0	6	12.667	0.023
Mouse	28.444	843.315	0.531	15.757	8433150	157574	2780048	3.033	0.006
P22	2.476	73.415	0.047	1.381	734152	13806	42	17479.810	31.874
ϕVPE25	1.888	55.971	0.041	1.227	559712	12270	85	6584.847	12.007
ϕX174	0.414	12.265	6 E-5	0.002	122648	18	5	24529.600	44.730
P. phaseolicola HB10Y	1 E-5	2 E-4	0	0	2	0	6200	3 E-4	6 E-7
S. typhimurium LT2	0.043	1.270	0.003	0.086	12704	864	5022	2.530	0.005
T3	0.159	4.708	0.004	0.107	47084	1070	38	1239.053	2.259
T7	0.248	7.354	0.021	0.628	73538	6278	40	1838.450	3.352

Table S10. Details on read mapping, sample MG2

Organism	*% Unambiguous reads*	*Unambiguous MB*	*% Ambiguous reads*	*Ambiguous MB*	*Unambiguous reads*	*Ambiguous reads*	*Genome size (kbp)*	*Unambigious reads/genome size*	*% Normmalized to genome size*
B. thetaiotaomicron VPI5482	51.498	1924.723	0.698	26.097	19247228	260968	6383	3015.389	6.344
E. coli B	0.001	0.029	0.009	0.332	286	3320	4624	0.062	1 E-4
E. faecalis V583	0	0	0	0.004	0	36	3408	0	0
Human	0.014	0.517	0.008	0.307	5170	3066	3209286	0.002	3 E-6
L. monocytogenes EGD-e	2.706	101.151	0.060	2.242	1011508	22418	2986	338.750	0.713
M13	2 E-4	0.009	0	0	90	0	6	15.000	0.032
Mouse	40.806	1525.138	0.707	26.408	15251380	264080	2780048	5.486	0.012
P22	1.432	53.507	0.039	1.455	535068	14554	42	12739.714	26.802
ϕVPE25	0.810	30.264	0.027	1.026	302642	10260	85	3560.494	7.491
ϕX174	0.344	12.850	6 E-5	0.002	128496	24	5	25699.200	54.066
P. phaseolicola HB10Y	1 E-5	2 E-4	0	0	2	2	6200	3 E-4	7 E-7
S. typhimurium LT2	0.024	0.903	0.002	0.061	9032	608	5022	1.798	0.004
T3	0.094	3.508	0.003	0.114	35078	1140	38	923.105	1.942
T7	0.132	4.936	0.012	0.463	49360	4630	40	1234.000	2.596

Table S11. Plaque forming unit (PFU) count versus virus-like particle (VLP) count for dsDNA phages in phage stocks used to prepare the artificial microbiome sample

Phage	*VLP / PFU ratio*	*Ratio of phage input into artificial microbiome sample based on VLP counts*
P22	4.8	1.0
ϕVPE25	5.7	1.2
T3	7.2	1.5
T7	8.2	1.7
